# Molecular point-of-care testing for respiratory viruses versus routine clinical care in adults with acute respiratory illness presenting to secondary care: a pragmatic randomised controlled trial protocol (ResPOC)

**DOI:** 10.1186/s12879-017-2219-x

**Published:** 2017-02-06

**Authors:** Nathan J. Brendish, Ahalya K. Malachira, Tristan W. Clark

**Affiliations:** 1 NIHR Southampton Wellcome Trust Clinical Research Facility, University Hospital Southampton NHS Foundation Trust, Southampton, UK; 2 Clinical and Experimental Sciences, Faculty of Medicine, University of Southampton, Southampton, UK; 3 NIHR Southampton Respiratory Biomedical Research Unit, University Hospital Southampton NHS Foundation Trust, Southampton, UK

**Keywords:** Point-of-care test, Influenza, Respiratory virus, Adult, Hospitalised, Acute respiratory illness

## Abstract

**Background:**

Respiratory viruses are associated with a huge socio-economic burden and are responsible for a large proportion of acute respiratory illness in hospitalised adults. Laboratory PCR is accurate but takes at least 24 h to generate a result to clinicians and antigen-based point-of-care tests (POCT) lack sensitivity. Rapid molecular platforms, such as the FilmArray Respiratory Panel, have equivalent diagnostic accuracy to laboratory PCR and can generate a result in 1 h making them deployable as POCT. Molecular point-of-care testing for respiratory viruses in hospital has the potential to improve the detection rate of respiratory viruses, improve the use of influenza antivirals and reduce unnecessary antibiotic use, but high quality randomised trials with clinically relevant endpoints are needed.

**Methods:**

The ResPOC study is a pragmatic randomised controlled trial of molecular point-of-care testing for respiratory viruses in adults with acute respiratory illness presenting to a large teaching hospital in the United Kingdom. Eligible participants are adults presenting with acute respiratory illness to the emergency department or the acute medicine unit. Participants are allocated 1:1 by internet-based randomisation service to either the intervention of a nose and throat swab analysed immediately on the FilmArray Respiratory Panel as a POCT or receive routine clinical care. The primary outcome is the proportion of patients treated with antibiotics. Secondary outcomes include turnaround time, virus detection, neuraminidase inhibitor use, length of hospital stay and side room use. Analysis of the primary outcome will be by intention-to-treat and all enrolled participants will be included in safety analysis.

**Discussion:**

Multiple novel molecular POCT platforms for infections including respiratory viruses have been developed and licensed in the last few years and many more are in development but the evidence base for clinical benefit above standard practice is minimal. This randomised controlled trial aims to close this evidence gap by generating high quality evidence for the clinical impact of molecular POCT for respiratory viruses in secondary care and to act as an exemplar for future studies of molecular POCT for infections. This study has the potential to change practice and improve patient care for patients presenting to hospital with acute respiratory illness.

**Trial registration:**

This study was registered with ISRCTN, number ISRCTN90211642, on 14th January 2015.

## Background

### Background and rationale

#### Respiratory virus burden of disease

Respiratory tract infections are the second most common cause of mortality and morbidity worldwide [1] and viruses are the most frequently detected pathogens in acute respiratory illness [2]. The influenza virus causes seasonal epidemics leading to excess hospitalisations and death mainly in the elderly and in patients with co-morbidity [3, 4]. Annual seasonal influenza vaccine is recommended in at risk groups [5–7] however vaccine coverage rate is sub-optimal [8, 9] and high quality evidence for significant protection in the elderly is lacking [10, 11].

The rate of hospitalisation in adults with influenza has been estimated at 5 to 20 per 100,000 overall [12, 13] and may be as high as 1,200 per 100,000 in those over 85 years old [4]. Hospitalisation and death result from the complications of influenza including pneumonia and exacerbation of underlying cardiopulmonary conditions [14]. In adult patients hospitalised with laboratory confirmed influenza, 10–30% are admitted to critical care units and 3–15% die in hospital [15–17] with outcomes being predicted by co-morbidity [17, 18]. Estimates of the burden of influenza virus infection in hospitalised adults have traditionally been based on the incidence of the influenza-like-illness syndrome (ILI, defined as fever of >38 °C and new respiratory symptoms) rather than on laboratory confirmed influenza. ILI has poor sensitivity (around 50%) and specificity (0–63%) for the diagnosis of influenza in hospitalised adults even during periods of peak activity [19–22]. Where estimates of disease burden are based on laboratory confirmed influenza, laboratory testing of patients is based on clinical suspicion of influenza and is generally targeted to patients with respiratory symptoms and fever. However, in addition to acute respiratory presentations, influenza may present as decompensated cardiovascular disease, collapse or diabetic emergencies [23, 24]. For this reason many hospitalised cases of influenza are likely to remain undiagnosed. A recent Canadian study estimated that only around one in 14 ED visits due to influenza virus infection were correctly attributed to influenza [25]. It is likely, therefore, that the burden of influenza and other respiratory viruses amongst hospitalised adults and its economic impact have been under-estimated. In addition to influenza viruses, other respiratory viruses including rhinovirus, respiratory syncytial virus, parainfluenza viruses, human metapneumovirus and coronaviruses, cause acute exacerbations of COPD and asthma as well as other acute respiratory presentations [2], which lead to large numbers of hospitalisations every year and significant burdens upon healthcare systems.

#### Conventional rapid diagnostic tests

Rapid diagnostic tests for influenza, and other respiratory viruses, based on antigen detection in nasal samples have been available for many years but have been diagnostically inaccurate in adults, where sensitivity is around 50% [26, 27]. The poor sensitivity of these antigen-based tests limits their clinical utility and their use has not been associated with a reduction in antibiotic prescribing in adults [28].

The current gold standard diagnostic test for respiratory viruses is laboratory performed polymerase chain reaction (PCR) which is highly sensitive and specific but has turnaround times of at least 24 h and requires specialist laboratory facilities and expertise [29]. New rapid, molecular tests have recently been developed, including the FilmArray Respiratory Panel. These molecular platforms are comparable in accuracy to laboratory PCR, without the need for specialist laboratory support and expertise, and can potentially be used as a point-of-care test (POCT), but the evidence for molecular POCT improving patient outcomes is weak.

#### FilmArray Respiratory Panel

The FilmArray Respiratory Panel (BioFire Diagnostics, Utah, USA, owned by bioMérieux) uses nested real-time PCR to detect 20 respiratory pathogens. The FilmArray requires only 2 min of “hands on” time and produces a test result in about one hour [30]. The FilmArray Respiratory Panel is both FDA-cleared and CE IVD marked. The viral pathogens detected by the FilmArray Respiratory Panel are: Influenza A (untyped, A/H1, A/H1-2009, A/H3), Influenza B, Adenovirus, Coronaviruses (HKU1, NL63, 229E, OC43) Human Metapneumovirus, Human Rhinovirus/Enterovirus, Parainfluenza (types 1, 2, 3, 4) and Respiratory Syncytial Virus. Three bacterial respiratory pathogens are also detected: *Bordetella pertussis*, *Chlamydophila pneumoniae* and *Mycoplasma pneumoniae* [30, 31].

The FilmArray respiratory panel is broadly equivalent in accuracy to laboratory PCR, and use has been validated on nose and throat swabs, nasopharyngeal aspirates, lower respiratory tract samples, and samples from immunocompromised patients [31–37]. Initial mediocre sensitivity for adenovirus detection has been greatly improved [38]. All of these studies show favourable outcomes with FilmArray system, including reliability, accuracy, ease of use and turnaround time although these studies were conducted in a laboratory rather at the point-of-care and a disproportionate number of these studies were conducted from samples from children rather than adults.

#### Potential clinical impact of point-of-care testing for respiratory viruses

In hospitalised adults with acute respiratory illness, viruses are the most commonly detectable pathogen, with bacterial detection being much less frequent, although antibiotic use is almost universal [2]. Antibiotic over-use is partly driven by clinical uncertainty as to the aetiology of the acute respiratory illness and so by early identification of viruses antibiotic use may be prevented.

A study examining clinical outcomes in children has shown that use of the FilmArray reduced the duration of antibiotic use, the length of inpatient stay, and the time in isolation [39]. However, this was not a randomised controlled trial but examined outcomes pre- and post-implementation. To our knowledge, there have been no randomised controlled trials of this system as a point-of-care test examining the potential clinical and health economic benefits. Although there are data to suggest clinical benefits (in terms of duration of hospital stay, number of investigations, and antibiotic use) for use of rapid diagnostic tests of influenza and other respiratory viruses in children [40–43], the clinical benefits and cost effectiveness of such a strategy in adults are unknown [44].

A Cochrane review evaluating the use of rapid antigen tests for acute respiratory illness in children in the emergency department concluded that there is currently insufficient evidence to support rapid viral to reduce antibiotic use in this setting. The authors suggest an adequately powered trial with antibiotic use as the primary outcome [45].

#### Point-of-care testing in the wider context

The Department of Health commissioned Carter report into UK pathology services noted the importance of developing clinically relevant point-of-care diagnostic tests to reduce turnaround times and improve patient pathways [46]. The UK Medicines and Healthcare products Regulatory Agency (MHRA) document ‘Management and Use of Point-of-Care Test Devices’ sets out the context in which POCT should be considered for use and provides guidelines for their successful and safe implementation [47]. The objectives of this study are in line with these documents and it aims to examine the initial phase of a POCT programme: establishing a clinical need for the test and evaluating potential clinical benefits.

#### Alignment with global research priorities

In addition to being focused on patient and healthcare organisation outcomes, this clinical research is strongly aligned with several global research priority initiatives including the World Health Organisation’s Battle against Respiratory Viruses (BRaVe) initiative [48] and the global report into antibiotic resistance [49]. The BRaVe initiative aims to catalyse multidisciplinary research on strategies to prevent and treat medically important respiratory virus infections with the goal of timely integration of research advances into public health practice. Priority areas identified include improving diagnostic tests for viral respiratory illness and improving the clinical management of patients with acute respiratory viral illness which are both addressed by this study.

### Study aims and objectives

This study aims to prospectively evaluate whether use of a molecular point-of-care diagnostic test will improve clinical outcomes compared to routine clinical care, in adult patients presenting to the secondary care with acute respiratory illness. The primary objective of the study is to evaluate the impact of POCT on antibiotic use. The secondary objectives include evaluating the impact of POCT on influenza antiviral use, side room facility use, duration of hospitalisation and the turnaround time of results compared with standard laboratory based PCR.

### Trial design

This is a pragmatic randomised controlled trial with parallel groups allocated 1:1 to the intervention (POCT) and control (routine clinical care) arms. The framework is superiority. The study protocol adheres to the SPIRIT statement [50].

## Methods

### Participants, interventions and outcomes

#### Study setting

This is a single centre study based in secondary care. All patients will be recruited from the Acute Medical Unit (AMU) and Emergency Department (ED) of Southampton General Hospital, University Hospital Southampton NHS Foundation Trust, Southampton, UK.

#### Eligibility criteria

##### Inclusion criteria

- Aged 18 years or over
- Has the capacity to give informed, written consent and is able and willing to adhere to the study procedures
- Is a patient in Southampton General Hospital’s AMU or ED
- Can be recruited to the study
  - within a 24 h period of first triage by ED staff OR
  - within a 24 h period of arrival on AMU (if admitted directly to AMU)
- Has an acute respiratory illness* and/or fever >37.5 °C
- Duration of illness less than or equal to 7 days

*An episode of acute respiratory illness is defined as an acute pulmonary illness (including pneumonia, bronchitis and influenza-like illness) or an acute exacerbation of a chronic respiratory illness (including exacerbation of COPD, asthma or bronchiectasis).

Provisional or suspected clinical diagnoses of acute respiratory illnesses are made by an AMU or ED clinician.

##### Exclusion criteria

- Patients not fulfilling inclusion criteria
- A palliative approach being taken by the treating clinicians
- Previously included in this study and re-presenting within the last 30 days after hospital discharge
- Declines nasal/pharyngeal swabbing

Concurrent, prior or subsequent enrolment in an observational study is not necessarily an exclusion criterion; this is at the discretion of the chief investigator.

#### Interventions

##### For those randomised to the interventional arm

A nose and throat swab will be taken by a member of research staff (doctor or nurse) according to standard protocols. Combined nose and throat swabs have been described as an effective approach to maximise PCR sensitivity for a large number of respiratory viruses [51]. Swabs are placed directly into viral transport medium (Sigma Virocult®, MWE, UK). The sample is analysed on the FilmArray Respiratory Panel as per training delivered by the apparatus manufacturer. Test results are normally available in about an hour using the FilmArray Respiratory Panel. In the event of a run failure, the analysis run will be repeated using the same sample. The FilmArray machines are located in or near the patient-care areas (AMU and ED). The machines may be operated by a study doctor, research nurse or research technician. The results of the test will be documented in the patient’s case notes and in the event of a pathogen being detected, a doctor from the clinical team responsible for the patient will be directly informed. The participant will also be informed of the result on the same day.

##### For those randomised to the control group

These patients will be managed using routine clinical care, as per current practice in this large teaching hospital in the United Kingdom, which is a justifiable comparator. Respiratory virus testing using laboratory PCR will be at the discretion of the responsible clinical team.

##### For both groups

A subgroup of participants may be approached for venous blood sampling and additional nasal/throat swabs to be stored for further study including immunological testing and viral sequencing. All samples will be stored devoid of participant identifiable information to protect participant confidentiality.

#### Outcomes

##### Primary outcome

- Proportion of patients treated with antibiotics, measured retrospectively from case notes for the entire duration of hospitalisation or at 30 days, whichever is shortest

##### Secondary outcomes

- Median duration of antibiotic use, days
- Proportion of patients receiving only a stat dose of antibiotics
- Proportion of patients receiving <48 h antibiotics
- Proportion of patients receiving intravenous antibiotics
- Median duration of intravenous antibiotics, days
- Proportion of patients with influenza treated with influenza antivirals
- Proportion of influenza antiviral use occurring in patients with influenza
- Median time to influenza antiviral use, hours
- Median duration of influenza antivirals, days
- Median duration of hospital stay, days
- Proportion of patients admitted to a side room*
- Median duration of side room use, days*
- Median time to isolation or de-isolation, days*
- Median length of hospital stay, days
- Median turnaround time of respiratory viruses testing, hours
- Proportion of patients with viruses detected
- Proportionate mortality in hospital and at 30 days post randomisation
- Proportion admitted to Intensive care or high dependency units
- Proportion re-presenting to hospital within 30 days
- Proportion re-admitted to hospital within 30 days
- Proportion with prolonged in-patient stay

* A side room is a single-patient room; the use of which is a surrogate measure for isolation for the purpose of infection prevention and control.

Every attempt will be made to recruit patients and deliver the results of POCT prior to antibiotic use however it is understood due to the usual processes of care in hospital that on some occasions antibiotics will be have been administered before POCT is performed or before results are available to clinical teams. Therefore we plan to also undertake a *post hoc* analysis of only those patients where antibiotics were not administered prior to POCT results being available to clinicians.

In view of the deliberately broad inclusion criteria, heterogeneity of treatment effect among different clinical groups is anticipated and therefore subgroup analysis for the primary and certain key secondary outcome measures is planned based on clinical group. Results for antibiotic use (including any use, single dose, and use of less than 48 h duration), duration of antibiotics, admission and length of hospitalisation will be assessed separately for each clinical subgroup.

#### Participant timeline

Patients are identified in the AMU and ED by research staff according to eligibility criteria and once written informed consent is obtained they are immediately randomised to the intervention or control group. Those randomised to the intervention group have a nose and throat swab performed immediately by a study doctor or research nurse and this is then tested on the FilmArray machine. Results are available after approximately 1 h and are immediately communicated to the clinical team. Clinical data is then collected retrospectively for both groups. There are no follow up visits for either group.

#### Sample size

Sample size is based upon the primary outcome measure of proportion of patients treated with antibiotics. Previous studies have demonstrated that around 75% of patients hospitalised in the UK with acute respiratory illness are treated with antibiotics [2]. Two small studies in hospitalised adult patients with acute respiratory illness have demonstrated reductions in the proportion of patients treated with antibiotics of around 10–15% in those tested for respiratory viruses [44, 52]. To detect a reduction in antibiotic use from 75 to 65% with a power of 0.8 and significance level of 0.05, 326 patients would be required in each group. Allowing for withdrawals in up to 10% of patients we aim to recruit 360 patients to each group (720 patients in total).

#### Recruitment and screening

Eligible patients in the emergency department (ED) and acute medicine unit (AMU) of Southampton General Hospital will be identified by research staff who will regularly review the comprehensive IT admissions systems in each area on a daily basis. Recruitment will run from January 2015 until April 2015 and from October 2015 to April 2016 in order to include the periods of peak influenza circulation for those seasons.

### Assignment of interventions

#### Sequence generation, allocation concealment and implementation

Once an eligible patient has been screened, and given fully informed, written consent they will be enrolled and assigned a unique participant identification number consecutively. A study team member will then use a dedicated internet-based randomisation service (sealedenvelope.com, which uses random permuted blocks of varying sizes) to obtain a computer generated randomisation code for the patient which will assign them to either the intervention or control group. Research staff will implement the allocation sequence and assign the patients to the group based on the allocation code from sealedenvelope.com.

#### Blinding

As this is a pragmatic trial of a diagnostic device no attempt at blinding trial participants, research staff or care providers will be made. Data analysts will be blinded to group allocation.

### Data collection, management and analysis

#### Data collection methods

Clinical and demographic data will be collected at the time of enrolment by research staff from patient paper case notes and electronic medical records. Outcome data will be collected retrospectively by research staff from paper case notes, electronic medical records, electronic prescribing systems, and electronic radiological and laboratory results systems. Final clinical diagnosis will be based on clinical discharge coding and discharge summaries. All source data will be entered into a standardised paper case report form. Patients withdrawn from the study will have no further data collected. Missing data was minimal in the pilot study and is not expected to be a significant issue; the use of multiple imputation will be considered should missing data exceed 10% in the primary outcome.

#### Data management

The study will be conducted in accordance with the approved protocol, ICH GCP relevant regulations and standard operating procedures. Data will be evaluated for compliance with the protocol and accuracy in relation to source documents. Data from case report forms will be entered into a secure bespoke database at the completion of the study followed by data lock. All data will be anonymised: volunteer participant data will be identified by a unique study number in the CRF and database. A separate confidential file containing identifiable information will be stored in a secured location in accordance with the Data Protection Act 1998. Only the Sponsor’s representative and investigators will have access to the information.

#### Statistical methods

This will be performed by a dedicated medical statistician from the University of Southampton independent from the study team. Patients tested with the rapid diagnostic test will be compared with patients treated by routine clinical care using standard descriptive and comparative statistical methods using Prism (GraphPad Software Inc; La Jolla, California) and SPSS (SPSS, Inc; Chicago, Illinois). Summaries of all baseline characteristics will be presented using means and standard deviations, medians and interquartile ranges, or frequencies and percentages, as appropriate. Analysis of the primary outcome will be by intention-to-treat and will compare the proportion of patients receiving antibiotics using the chi-square test for equality of proportions between groups. The effect of group (intervention or control) on the primary outcome will be further assessed using logistic regression to control for demographics (age, sex) and other co-variables. For secondary outcomes the intervention and control groups will be compared using chi-square tests for equality of proportions for binary data (including proportions) and using t-tests and non-parametric equivalent tests for continuous data (e.g. turnaround time) as appropriate.

### Monitoring

#### Data monitoring

The study was reviewed by the sponsor and felt to be of low risk on the grounds that it is not a clinical trial of an investigational medicinal product and the low likelihood of harms associated with the intervention. Therefore the creation of a data monitoring committee was not felt necessary. No interim analysis of data is planned.

#### Harms

The risks of nose and throat swabs and additional blood tests being taken are minimal and where occurring are likely to be mild. No additional adverse events s related to POCT for respiratory viruses are anticipated. However active monitoring and reporting of severe adverse events will be undertaken. Serious adverse events (SAE) are defined here as:

- Death during admission or within 30 days of enrolment
- Admission to the intensive care unit
- Evidence of prolonged hospital stay
- New or persistent significant disability or incapacity
- Evidence of congenital anomaly or birth defect

As participants in ED are not yet hospitalised but have a reasonable likelihood of being admitted to hospital, patients enrolled in ED who are subsequently admitted to the hospital will not automatically be counted as having experienced a SAE. Participants who are already admitted to AMU are already hospitalised however, an adverse event leading to prolongation of their existing hospitalisation will be counted as an SAE.

#### Auditing

Regular monitoring will be performed according to ICH GCP by the sponsor. Data will be evaluated for compliance with the protocol and accuracy in relation to source documents. Following written standard operating procedures, the monitors will verify that the clinical trial is conducted and data are generated, documented and reported in compliance with the protocol, GCP and the applicable regulatory requirements.

### Protocol amendments

All protocol modifications were communicated to investigators and to trial registries. Two amendments to the protocol have been approved by the ethics committee, the first, to change the study from a pilot study into a full study and to amend an exclusion criterion, the second, to add a laboratory analysis plan for the samples collected (current protocol version 3.0, date 26th January 2016). The local study reference is RHM MED1217.

### Confidentiality

All data will be anonymised to protect participant confidentiality: volunteer participant data will be identified by a unique study number in the case report forms and database. Serious Adverse Events will be reported in line with Good Clinical Practice and regulatory requirements. All study staff are trained in Good Clinical Practice. Only the investigators and sponsor’s representative (monitor) have access to the data, which is kept securely.

### Access to data

The final data set will be wholly accessible to the principal investigator, co-investigators and independent statistician and may be made available to other parties on request.

### Dissemination policy

Authorship of this and subsequent manuscripts stemming from this protocol will follow the ICMJE recommendations, and CONSORT statement where appropriate, and there is no intent to use professional writers. There are no plans to make the dataset publically available. Beyond the study team and regulatory oversight, the full protocol is only made available at the discretion of the chief investigator. The data and samples collected are expected to form multiple publications, and these publications must acknowledge this trial and study team as appropriate.

## Discussion

We hypothesise that molecular point-of-care testing for respiratory viruses, in adults presenting to hospital with acute respiratory infection, will reduce antibiotic use, improve detection of influenza and use of neuraminidase inhibitors, improve isolation facility use and be safe.

This study has the potential to improve patient care by changing practice and contribute to a health economic analysis. The outcome measures are clinically important to a large number of patients, and also crucial in antimicrobial stewardship and healthcare resource management. As a randomised controlled trial, this study will provide high-quality evidence for the potential use of molecular point-of-care testing for respiratory viruses in hospitalised adults. Beyond this trial, molecular point-of-care testing for common pathogens in select populations, such as in intensive care, or other common illness presentations, such as gastroenteritis, needs to be evaluated to further improve patient care and effectively manage healthcare resources.

## Abbreviations

AMU: Acute medicine unit
BRaVe: Battle against Respiratory Viruses (WHO initiative)
ED: Emergency department
GCP: Good Clinical Practice
ICH: International Conference on Harmonisation
ILI: Influenza-like illness
PCR: Polymerase chain reaction
POCT: Point-of-care test
SAE: Serious adverse event

## References

[1] Lozano R, Naghavi M, Foreman K, Lim S, Shibuya K, Aboyans V (2012). Global and regional mortality from 235 causes of death for 20 age groups in 1990 and 2010: a systematic analysis for the Global Burden of Disease Study 2010. Lancet.

[2] Clark TW, Medina MJ, Batham S, Curran MD, Parmar S, Nicholson KG (2014). Adults hospitalised with acute respiratory illness rarely have detectable bacteria in the absence of COPD or pneumonia; viral infection predominates in a large prospective UK sample. J Infect.

[3] Thompson WW, Shay DK, Weintraub E, Brammer L, Cox N, Anderson LJ (2003). Mortality associated with influenza and respiratory syncytial virus in the United States. JAMA.

[4] Thompson WW, Shay DK, Weintraub E, Brammer L, Bridges CB, Cox NJ (2004). Influenza-associated hospitalizations in the United States. JAMA.

[5] van Essen GA, Palache AM, Forleo E, Fedson DS (2003). Influenza vaccination in 2000: recommendations and vaccine use in 50 developed and rapidly developing countries. Vaccine.

[6] Fiore AE, Uyeki TM, Broder K, Finelli L, Euler GL, Singleton JA (2010). Prevention and control of influenza with vaccines: recommendations of the Advisory Committee on Immunization Practices (ACIP), 2010. MMWR Recomm Rep.

[7] World Health Organisation (WHO) (2012). Regional Office for Europe: WHO/Europe recommendations on influenza vaccination during the 2011/2012 Winter Season.

[8] Blank PR, Schwenkglenks M, Szucs TD (2009). Vaccination coverage rates in eleven European countries during two consecutive influenza seasons. J Infect.

[9] Lu PJ, Santibanez TA, Williams WW, Zhang J, Ding H, Bryan L (2013). Surveillance of influenza vaccination coverage—United States, 2007–08 through 2011–12 influenza seasons. MMWR Surveill Summ.

[10] Osterholm MT, Kelley NS, Sommer A, Belongia EA (2012). Efficacy and effectiveness of influenza vaccines: a systematic review and meta-analysis. Lancet Infect Dis.

[11] Simonsen L, Taylor RJ, Viboud C, Miller MA, Jackson LA (2007). Mortality benefits of influenza vaccination in elderly people: an ongoing controversy. Lancet Infect Dis.

[12] Dao CN, Kamimoto L, Nowell M, Reingold A, Gershman K, Meek J (2010). Adult hospitalizations for laboratory-positive influenza during the 2005–2006 through 2007–2008 seasons in the United States. J Infect Dis.

[13] Widmer K, Zhu Y, Williams JV, Griffin MR, Edwards KM, Talbot HK (2012). Rates of hospitalizations for respiratory syncytial virus, human metapneumovirus, and influenza virus in older adults. J Infect Dis.

[14] Irwin DE, Weatherby LB, Huang WY, Rosenberg DM, Cook SF, Walker AM (2001). Impact of patient characteristics on the risk of influenza/ILI-related complications. BMC Health Serv Res.

[15] Gaunt ER, Harvala H, McIntyre C, Templeton KE, Simmonds P (2011). Disease burden of the most commonly detected respiratory viruses in hospitalized patients calculated using the disability adjusted life year (DALY) model. J Clin Virol.

[16] Mauskopf J, Klesse M, Lee S, Herrera-Taracena G (2013). The burden of influenza complications in different high-risk groups: a targeted literature review. J Med Econ.

[17] Li G, Yilmaz M, Kojicic M, Fernández-Pérez E, Wahab R, Huskins WC (2009). Outcome of critically ill patients with influenza virus infection. J Clin Virol.

[18] Meier CR, Napalkov PN, Wegmüller Y, Jefferson T, Jick H (2000). Population-based study on incidence, risk factors, clinical complications and drug utilisation associated with influenza in the United Kingdom. Eur J Clin Microbiol Infect Dis.

[19] van den Dool C, Hak E, Wallinga J, van Loon AM, Lammers JW, Bonten MJ (2008). Symptoms of influenza virus infection in hospitalized patients. Infect Control Hosp Epidemiol.

[20] Babcock HM, Merz LR, Dubberke ER, Fraser VJ (2008). Case-control study of clinical features of influenza in hospitalized patients. Infect Control Hosp Epidemiol.

[21] Babcock HM, Merz LR, Fraser VJ (2006). Is influenza an influenza-like illness? Clinical presentation of influenza in hospitalized patients. Infect Control Hosp Epidemiol.

[22] Call SA, Vollenweider MA, Hornung CA, Simel DL, McKinney WP (2005). Does this patient have influenza?. JAMA.

[23] Falsey AR, Hennessey PA, Formica MA, Cox C, Walsh EE (2005). Respiratory syncytial virus infection in elderly and high-risk adults. N Engl J Med.

[24] Warren-Gash C, Smeeth L, Hayward AC (2009). Influenza as a trigger for acute myocardial infarction or death from cardiovascular disease: a systematic review. Lancet Infect Dis.

[25] Schanzer DL, Schwartz B (2013). Impact of seasonal and pandemic influenza on emergency department visits, 2003–2010, Ontario, Canada. Acad Emerg Med.

[26] Centres for Disease Control and Prevention (CDC). Evaluation of rapid influenza diagnostic tests for detection of novel influenza A (H1N1) Virus–United States, 2009. MMWR Morb Mortal Wkly Rep. 2009;58:826–9. 19661856

[27] Chartrand C, Leeflang MM, Minion J, Brewer T, Pai M (2012). Accuracy of rapid influenza diagnostic tests: a meta-analysis. Ann Intern Med.

[28] Nicholson KG, Abrams KR, Batham S, Medina MJ, Warren FC, Barer M (2014). Randomised controlled trial and health economic evaluation of the impact of diagnostic testing for influenza, respiratory syncytial virus and Streptococcus pneumoniae infection on the management of acute admissions in the elderly and high-risk 18- to 64-year-olds. Health Technol Assess.

[29] Mackay IM (2004). Real-time PCR, in the microbiology laboratory. Clin Microbiol Infect.

[30] BioFire Diagnostics. FilmArray® Respiratory Panel Information Sheet. http://www.biofiredx.com/support/documents/. Accessed 26 Jan 2017.

[31] Popowitch EB, O’Neill SS, Miller MB (2013). Comparison of the Biofire FilmArray RP, Genmark eSensor RVP, Luminex xTAG RVPv1, and Luminex xTAG RVP fast multiplex assays for detection of respiratory viruses. J Clin Microbiol.

[32] Poritz MA, Blaschke AJ, Byington CL, Meyers L, Nilsson K, Jones DE (2011). FilmArray, an automated nested multiplex PCR system for multi-pathogen detection: development and application to respiratory tract infection. PLoS ONE.

[33] Butt SA, Maceira VP, McCallen ME, Stellrecht KA (2014). Comparison of three commercial RT-PCR systems for the detection of respiratory viruses. J Clin Virol.

[34] Pierce VM, Elkan M, Leet M, McGowan KL, Hodinka RL (2012). Comparison of the Idaho Technology FilmArray system to real-time PCR for detection of respiratory pathogens in children. J Clin Microbiol.

[35] Loeffelholz MJ, Pong DL, Pyles RB, Xiong Y, Miller AL, Bufton KK (2011). Comparison of the FilmArray Respiratory Panel and Prodesse Real-Time PCR Assays for Detection of Respiratory Pathogens. J Clin Microbiol.

[36] Ruggiero P, McMillen T, Tang YW, Babady NE (2013). Evaluation of the BioFire FilmArray Respiratory Panel and the GenMark eSensor Respiratory Viral Panel on Lower Respiratory Tract Specimens. J Clin Microbiol.

[37] Hammond SP, Gagne LS, Stock SR, Marty FM, Gelman RS, Marasco WA (2012). Respiratory virus detection in immunocompromised patients with FilmArray respiratory panel compared to conventional methods. J Clin Microbiol.

[38] Doern CD, Lacey D, Huang R, Haag C (2013). Evaluation and Implementation of FilmArray Version 1.7 for Improved Detection of Adenovirus Respiratory Tract Infection. J Clin Microbiol.

[39] Rogers BB, Shankar P, Jerris RC, Kotzbauer D, Anderson EJ, Watson JR (2015). Impact of a rapid respiratory panel test on patient outcomes. Arch Pathol Lab Med.

[40] Noyola DE, Demmler GJ (2000). Effect of rapid diagnosis on management of influenza A infections. Pediatr Infect Dis J.

[41] Bonner AB, Monroe KW, Talley LI, Klasner AE, Kimberlin DW (2003). Impact of the rapid diagnosis of influenza on physician decision-making and patient management in the pediatric emergency department: results of a randomized, prospective, controlled trial. Pediatrics.

[42] Blaschke AJ, Shapiro DJ, Pavia AT, Byington CL, Ampofo K, Stockmann C (2014). A National Study of the Impact of Rapid Influenza Testing on Clinical Care in the Emergency Department. J Pediatric Infect Dis Soc.

[43] Mills JM, Harper J, Broomfield D, Templeton KE (2011). Rapid testing for respiratory syncytial virus in a paediatric emergency department: benefits for infection control and bed management. J Hosp Infect.

[44] Falsey AR, Murata Y, Walsh EE (2007). Impact of rapid diagnosis on management of adults hospitalized with influenza. Arch Intern Med.

[45] Doan Q, Enarson P, Kissoon N, Klassen TP, Johnson DW. Rapid viral diagnosis for acute febrile respiratory illness in children in the Emergency Department. Cochrane Database Syst Rev. 2014;(9):CD006452. doi:10.1002/14651858.CD006452.pub4. 10.1002/14651858.CD006452.pub4 PMC6718218 25222468

[46] Lord Carter of Coles (Chair) (2008). Report of the Second Phase of the Review of NHS Pathology Services in England.

[47] MHRA. Management and use of IVD point of care test devices. https://www.gov.uk/government/publications/in-vitro-diagnostic-point-of-care-test-devices. Accessed 26 Jan 2017.

[48] Legand A, Hayden FG (2013). Addressing the public health burden of respiratory viruses: the Battle against Respiratory Viruses (BRaVe) Initiative. Futur Virol.

[49] World Health Organisation (WHO) (2014). Antimicrobial resistance.

[50] Chan A-W, Tetzlaff JM, Altman DG, Laupacis A, Gøtzsche PC, Krleža-Jerić K (2013). SPIRIT 2013 Statement: Defining standard protocol items for clinical trials. Ann Intern Med.

[51] Kim C, Ahmed JA, Eidex RB, Nyoka R, Waiboci LW, Erdman D (2011). Comparison of Nasopharyngeal and Oropharyngeal Swabs for the Diagnosis of Eight Respiratory Viruses by Real-Time Reverse Transcription-PCR Assays. PloS ONE.

[52] Oosterheert JJ, van Loon AM, Schuurman R, Hoepelman AI, Hak E, Thijsen S (2005). Impact of rapid detection of viral and atypical bacterial pathogens by real-time polymerase chain reaction for patients with lower respiratory tract infection. Clin Infect Dis.

